# Commentary: Psychedelic Psychiatry's Brave New World

**DOI:** 10.3389/fpsyt.2020.594077

**Published:** 2020-11-26

**Authors:** Bénédicte Nobile, Emilie Olié, Philippe Courtet

**Affiliations:** ^1^Department of Emergency Psychiatry and Acute Care, Lapeyronie Hospital, CHU Montpellier, Montpellier, France; ^2^PSNREC, Univ Montpellier, INSERM, CHU de Montpellier, Montpellier, France; ^3^FondaMental Foundation, Créteil, France

**Keywords:** psilocybin, psychedelics, suicide, suicidal behavior, therapeutic

## Introduction

In their recent article entitled “Psychedelic Psychiatry's Brave New World,” Nutt et al. (1) wrote that new research on psychedelic drugs, especially psilocybin, has highlighted their potential for psychiatric disorder management. In the past decade, many studies focused on psilocybin effects on mood disorders (2) showing that it can significantly improve depressive symptomatology for weeks and even for years in some people (1). However, Nutt et al. (1) focused on depression and addiction, without mentioning the potential interest of psilocybin to prevent suicidal behavior (SB; i.e., completed suicide and suicide attempts) and to decrease suicidal ideation (SI). Unfortunately, SB and SI are often considered a symptom or a consequence of depression, although growing evidences indicate that SB does not overlap with depression and deserves specific attention (3). As SB is a major public health problem (more than 800,000 deaths by suicide/year worldwide, and 20 to 30 times more suicide attempts), still without specific and efficient treatments, it is urgent to find effective therapeutics. Here, we propose that besides its antidepressant effect, psilocybin antisuicidal properties should be tested. Indeed, in a large American epidemiological study (190,000 subjects), the use of psilocybin and other psychedelic drugs (e.g., LSD) was linked to a decrease of SB and SI, while other illicit drugs were associated with increased suicidal risk (4). In a recent open-label study, psilocybin intake was associated with a significant SI decrease at 1 and 2 weeks (5). We hypothesize that psilocybin biological effects might counteract the pathophysiological abnormalities observed in SB.

## Biological Pathways Implicated in SB on which Psilocybin Acts

Several studies reported alterations of the serotoninergic (e.g., serotonin depletion), glutamatergic (e.g., alteration of the kynurenine pathway), and inflammatory systems (e.g., increased levels of proinflammatory cytokines), as well as neuroplasticity [e.g., low levels of brain-derived neurotrophic factor (BDNF)] in SB (6). Psilocybin is rapidly metabolized into its active metabolite psilocin that is a serotoninergic agonist with high affinity for 5-HT2A receptors (5-HT2AR) (1). Psilocybin intake results in a rapid increase in 5-HT2AR activity that might lead to higher BDNF expression and consequently to major synaptic changes (7) and might promote neuroplasticity. Postmortem studies showed that 5-HT2AR availability is increased in the brain of suicide victims, possibly due to genetic variations or compensatory mechanisms (6). Therefore, psilocybin-positive effect on neuroplasticity and the serotoninergic system could contribute to compensate SB-related impairment of these systems. Second, similarly to ketamine, which reduces SI (6), psilocybin is a modulator of glutamatergic activity in prefrontal circuits. A common mechanism of action through modulation of glutamatergic transmission (leading to neuroplastic adaptations) could underlie the antisuicidal action of psilocybin and ketamine (2). Third, psilocybin intake might induce a decrease of inflammation because 5-HT2AR are also localized in immune system cells. Indeed, psilocybin might affect cell signaling within the immune system by stimulating anti-inflammatory pathways (7).

## Neuropsychological Pathways Implicated in SB on which Psilocybin Acts

In neuroimaging studies, psilocybin intake has been associated with disruption of the default mode network connectivity. This might enhance cognitive flexibility (2) and promote changes from avoidance (i.e., the tendency to avoid unpleasant thoughts or emotions) to acceptance by patients. It is assumed that in psychedelic therapy, cognitive flexibility, and promotion of acceptance increase in response to psilocybin intake, reinforced by the slogan: “trust, let go, and be open,” but could also continue for a long time after the treatment (8). This effect might be particularly relevant for suicidal patients who are characterized by altered decision-making, reduced cognitive flexibility, and poor problem-solving ability (6). Furthermore, SB could be considered the most extreme expression of experiential avoidance. Thus, similarly to Acceptance and Commitment Therapy (9), psilocybin might be useful in SB by promoting acceptance. Moreover, psilocybin increases inner peace, patience and good humor/play, interpersonal gaze, and compassion (7). This might promote well-being and prevent SB. SB is associated with reduced specific autobiographical memories (6), whereas psilocybin intake is accompanied by increased autobiographical memory. This effect is amplified when music is played during the session because it increases the drug-induced visual imagery (9). Finally, the response to psilocybin intake correlates with the induction of a “mystical” or “spiritual” experience. The 30-item Mystical Experience Questionnaire scores positively predicted psilocybin-related changes in attitudes, behavior, and well-being (2). This suggests that psilocybin stimulates spirituality, and this might protect from suicide (10).

## Discussion

Psilocybin actions on neurobiological systems involved in SB (e.g., serotoninergic system, neuroplasticity, cognitive, and psychological functioning) and preliminary results on its impact on SI should encourage more research on its antisuicidal effects and related mechanisms of action. Clinicians and researchers might be afraid of testing this drug in suicidal patients, particularly due to the risk of “bad trips” that may facilitate suicidal acts. However, suicidal patients should be included in clinical trials on psilocybin by putting in place an adapted protocol, as already done for other drugs, such as ketamine (e.g., to stay with the patient throughout the session, to assess the patient's family and social support, to prepare emergency plans with patients, to have close clinical monitoring) (3). SI reduction by psilocybin may last for several months or even years in some patients. Therefore, psilocybin use could be particularly interesting because SI is often recurrent and persistent and is a very strong predictor of future death by suicide. Finally, psilocybin has been recently designated by the U.S. Food and Drug Administration as a “breakthrough therapy” for treatment-resistant depression (2), stressing its potential for treating serious psychiatric disorders, such as SB. To conclude, although all mechanisms underlying psilocybin effects are not understood yet, these elements should encourage setting-up clinical trials or open label studies to test its efficacy in patients with SB.

## Author Contributions

PC supervised the redaction of the paper. BN performed bibliographic research and wrote the manuscript. EO actively revised and contributed to the redaction of the manuscript. All authors have contributed to the manuscript and have accepted the final version of the paper.

## Conflict of Interest

The authors declare that the research was conducted in the absence of any commercial or financial relationships that could be construed as a potential conflict of interest.
